# Enhanced Beetle Luciferase for High-Resolution Bioluminescence Imaging

**DOI:** 10.1371/journal.pone.0010011

**Published:** 2010-04-02

**Authors:** Yoshihiro Nakajima, Tomomi Yamazaki, Shigeaki Nishii, Takako Noguchi, Hideto Hoshino, Kazuki Niwa, Vadim R. Viviani, Yoshihiro Ohmiya

**Affiliations:** 1 National Institute of Advanced Industrial Science and Technology (AIST), Ikeda, Osaka, Japan; 2 Tsuruga Institute of Biotechnology, TOYOBO Co., Ltd., Tsuruga, Fukui, Japan; 3 Laboratório de Bioquímica e Biotecnologia de Sistemas Bioluminescentes, Universidade Federal de São Carlos, Campus de Sorocaba, Sorocaba, São Paulo, Brazil; New Mexico State University, United States of America

## Abstract

We developed an enhanced green-emitting luciferase (ELuc) to be used as a bioluminescence imaging (BLI) probe. ELuc exhibits a light signal in mammalian cells that is over 10-fold stronger than that of the firefly luciferase (FLuc), which is the most widely used luciferase reporter gene. We showed that ELuc produces a strong light signal in primary cells and tissues and that it enables the visualization of gene expression with high temporal resolution at the single-cell level. Moreover, we successfully imaged the nucleocytoplasmic shuttling of importin α by fusing ELuc at the intracellular level. These results demonstrate that the use of ELuc allows a BLI spatiotemporal resolution far greater than that provided by FLuc.

## Introduction

Bioluminescence reporters are widely used in various aspects of biological functions, such as gene expression, post-translational modification, and protein-protein interaction *in vitro* and *in vivo* [1], [2]. Recent advances in luciferase technology permit the quantitative visualization of gene expression at single-cell resolution by imaging its luminescence in real-time using a highly sensitive charged-coupled device (CCD) camera [3], [4]. Although fluorescence imaging techniques that use fluorescent proteins (e.g., green fluorescent protein (GFP) and its derivatives) as probes have greatly contributed to the advancement of cell biology studies, BLI is emerging as a new and sensitive approach for understanding cell physiology.

Luciferase (i.e., enzyme) emits light by oxidizing luciferin (i.e., substrate) in a specific manner. Among the possible luciferase/luciferin combinations, the beetle luciferase and D-luciferin (benzothiazole) pair is the best probe for long-term and non-invasive reporting of cellular events, as the luminescence generated by their reaction is highly quantitative and has an extremely low background, and luciferin is highly stable and permeates easily into cells or tissues. In addition, external light illumination, which in fluorescence-based methods causes phototoxic damage to cells and bleaches the probes by repetitive illumination, is not required for the luminescence reaction; thus, the characteristic properties of the beetle luciferase/luciferin enable longitudinal and quantitative BLI. However, the light output of the luciferases currently available, which include FLuc from living cells, is insufficient for analyses at higher temporal and/or spatial resolution. In particular, BLI at the subcellular level is difficult because of the insufficient light output of available probes. A brighter luminescent probe is therefore required to improve the sensitivity and resolution of BLI at single-cell and intracellular resolution. To overcome the technical limitations of luciferase technology in BLI, we have developed an enhanced beetle luciferase from the previously cloned Brazilian click beetle *Pyrearinus termitilluminans* luciferase, which emits green light (λmax = 538 nm) with D-luciferin, and whose emission color is pH-insensitive [5], by optimizing its cDNA sequence for mammalian expression.

## Results and Discussion

### Improvement of *P. termitilluminans* green-emitting luciferase for mammalian expression

*Pyrearinus termitilluminans* luciferase displays the most blue-shifted spectrum among the beetle luciferases [5]. Recently the purified enzyme has been shown to display high catalytic properties and thermostability *in vitro* among the pH-insensitive luciferases, producing to bright signals [6]. To improve the expression of *P. termitilluminans* luciferase in mammalian cells, we optimized codons of its cDNA sequence for mammalian expression and deleted putative transcription factor binding sites within the cDNA, without changing the deduced amino acid sequence (Figure S1). We refer to this luciferase as ELuc. We first compared the expression levels and bioluminescence intensities of the wild-type luciferase and the sequence-optimized luciferase, ELuc, in mouse fibroblast NIH3T3 cells (Figure S2A and B). The transient expression of ELuc in these cells under the control of the cytomegalovirus (CMV) promoter led to a dramatic increase in both the expression at the protein level and in the intensity of the bioluminescent signal (260-fold) in cell extracts when compared with those observed for wild-type luciferase. ELuc exhibits a bioluminescence spectrum in cell extracts with a peak at 538 nm that is identical to the wild-type form [5].

### Comparison of light output from FLuc and ELuc using a photomultiplier recording of clock gene expression in fibroblasts

**Figure 1:**
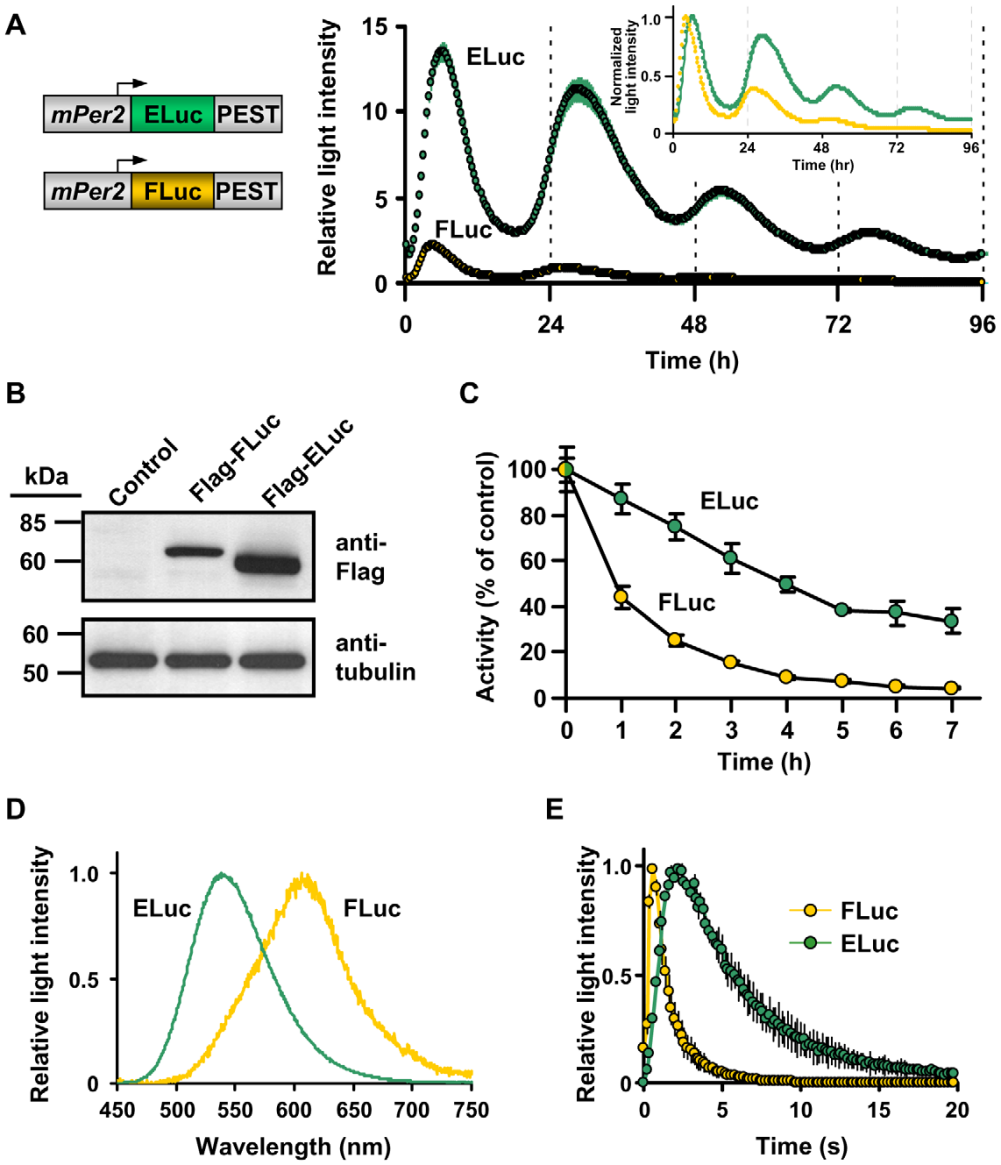
Comparison of the characteristic properties of FLuc and ELuc in NIH3T3 cells. (**A**) Photomultiplier recording of *mPer2* transcriptional oscillation in NIH3T3 cells expressing destabilized ELuc (green filled circles) and FLuc (orange filled circles). The reporter plasmids mPer2-dELuc or mPer2-dFLuc were cotransfected with pCMV-CLuc and cells were stimulated with 100 nM of dexamethasone. The respective luciferase activities were normalized to CLuc activity. Error bars indicate the standard deviation (n = 4). The *inset* shows recordings in the case that the peak values of the curves were set to 1. Schematic drawings of the reporter plasmids are shown on the left. (**B**) Western blot analysis of FLuc and ELuc in NIH3T3 cells. The expression plasmids pCMV-Flag::FLuc or pCMV-Flag::ELuc were transfected into NIH3T3 cells, which were harvested and disrupted 48 h later. Both luciferases were detected using the anti-Flag M2 antibody. Tubulin was used as an internal control. The positions of molecular weight markers are indicated on the left margin of each panel. (**C**) Stability of destabilized FLuc (orange filled circles) and ELuc (green filled circles) in NIH3T3 cells. NIH3T3 cells were independently transfected with SV40-dFLuc or SV40-dELuc and the culture medium was replaced with DMEM supplemented with 10% FBS and 100 µM cycloheximide. After 20 min (time = 0), incubation was continued in DMEM supplemented with 10% FBS and 100 µM cycloheximide. At the indicated times, cells were disrupted and luciferase activity was measured. Error bars indicate the standard deviation (n = 6). (**D**) Emission spectra of FLuc (orange line) and ELuc (green line) in viable cells. NIH3T3 cells seeded in 35 mm dishes were transfected with pGVC2 (for FLuc) or pCMV-ELuc and incubated for 48 h. To obtain the spectra, the culture medium was replaced with DMEM without phenol red supplemented with 10% FBS and 200 µM D-luciferin, and incubated for 12 h. Spectra were then measured. (**E**) Kinetics of light production by purified FLuc (orange filled circles) and ELuc (green filled circles) exposed to D-luciferin, ATP and Mg^2+^. The peak values were set to 1. Error bars indicate the standard errors (n = 3).

Next, we compared the light output of ELuc with that of FLuc from *Photinus pyralis* (*luc(+)*, Promega) in live cells. Both luciferases were fused with the PEST element of mouse ornithine decarboxylase and were expressed in NIH3T3 cells under the control of the mouse clock gene (*mPer2*) promoter using the same vector backbone. The bioluminescence of these molecules after stimulation with dexamethasone was recorded in real-time for 96 h in the presence of D-luciferin using a luminometer. Interestingly, although both luciferases monitored the circadian oscillation of *mPer2* with a slight delay in phase, ELuc produced a bioluminescent signal that was 14-fold higher than that of FLuc (Figure 1A). It is assumed that differences in the light output from cells may have been caused by differences in the level of expression and/or stability of the luciferases in these cells. The protein expression level of ELuc was 3-fold higher than that of FLuc, and the functional half-life of ELuc (t_1/2_ = 4 h) in NIH3T3 cells was significantly longer than that of FLuc (t_1/2_≤1 h) (Figure 1B and C), indicating that ELuc is highly expressed and moderately stable in the cells compared with FLuc.

### Measurement of the FLuc and ELuc spectra in viable cells

It has been reported that the emission spectrum of FLuc is influenced by reaction conditions, which include pH and temperature, with concomitant changes in quantum yield [7]-[11]. This property is probably also affected by the reaction condition in viable cells. In addition, the characteristic properties of the emission spectrum in living cells are a critically important feature in the multicolor luciferase assay method [12]-[14], in which the expression of multiple genes is monitored simultaneously using different color-emitting luciferases. We determined the emission spectra of ELuc and FLuc in NIH3T3 cell extracts and in living cells. ELuc exhibited a similar spectrum in cell extracts (data not shown) and in live cells (Figure 1D), with a peak at 538 nm. In contrast, FLuc exhibited a yellow-green light with a peak at 560 nm in cell extracts, as reported previously [7], [10], [11], whereas its peak was red-shifted to 610 nm (with a small shoulder at 560 nm) in living cells (Figure 1D), similar to *in vivo* observations [9]. Previous observations revealed that FLuc emits red light, the spectrum of which is similar to that observed in live cells (Figure 1D) at low pH or high temperature. In addition, the light output and the quantum yield drastically decrease under these conditions [8], [11]. These findings suggest that reaction efficiency, which includes the quantum yield of FLuc, was lower in live cells than in cell extracts, whereas the reaction efficiency of ELuc may be similar in the two contexts. Furthermore, the color of the stable bioluminescence produced by ELuc in live cells demonstrates the feasibility of its use in multicolor BLI in combination with other types of luciferases that emit at different wavelengths, whereas the sensitive spectral emission of FLuc is not amenable to this type of application.

### *In vitro* luminescence and kinetic measurements of purified FLuc and ELuc

To examine the difference in light output from FLuc and ELuc in living cells, we measured the luminescence intensity and kinetics of purified recombinant FLuc and ELuc. The emission spectra of both purified luciferases were consistent with those measured in cell extracts (data not shown). Figure S3A shows representative kinetics of the light output from purified FLuc and ELuc when PicaGene (Toyo Ink) was used as the luminescent substrate. After the addition of the substrate, the FLuc signal remained constant, whereas the ELuc signal gradually increased. The sustaining luminescence kinetics of FLuc are partly attributed to coenzyme A (CoA), a vestigial substrate of beetle luciferase included with the PicaGene reagent, by which CoA removes from the FLuc active site the inhibitors that are produced by luciferin–luciferase reaction [15], [16]. Similarly, CoA also seems to participate in the long-lasting light production of ELuc, as discussed by Silva Neto *et al.* [6]. In contrast to the light output from living cells, the resulting bioluminescence intensity of purified ELuc protein (normalized to the amount of protein) was unexpectedly slightly lower than that of FLuc, (Figure S3B), suggesting that the brighter luminescent signal from viable cells is not simply attributable to the enzymatic activity of the lucifearase.

Recently, several physicochemical properties of recombinant wild-type *P. termitilluminans* luciferase (the amino acid sequence of which is the same as that of ELuc, with the exception of a deletion of the duplicated first methionine, as described in the Materials and Methods section) have been determined, and these have been compared with those of other beetle luciferases, including FLuc [6]. The Michaelis–Menten constant (*K*_m_) of the wild-type *P. termitilluminans* luciferase for D-luciferin is rather higher than that of FLuc, whereas their *K*_m_ values for adenosine triphosphate (ATP) are almost identical. The catalytic constants of both luciferases are also similar. Therefore, these kinetic properties may not contributed to the high light output of ELuc in living cells. In contrast, the decay constant of the luminescence of the wild-type luciferase is higher than that of FLuc [6]. When we measured the luminescence kinetics of purified FLuc protein by adding ATP and Mg^2+^ as cofactors and D-luciferin, the luminescence displayed flash-like kinetics (Figure 1E), as descried previously [10]. In contrast, the purified ELuc protein displayed a low increase and a slow apparent decay rate compared with those of FLuc, suggesting a faster release of the luciferin–luciferase reaction product and slower inhibition by the product. In a preliminary study, we also measured the quantum yield of the purified ELuc protein and estimated it to be 1.5-fold higher than that of FLuc (unpublished data). Thus, the slow luminescence decay kinetics and the slightly higher quantum yield of ELuc should facilitated the producing of a stronger bioluminescent signal. However, we can not attribute the greater light intensity (over 10-fold) measured in living cells to these physicochemical properties alone. Therefore we assume that the higher expression of ELuc, achieved by the optimization of the cDNA sequence, and its stability in living cells contribute more to its much brighter signal in cells than do its physicochemical properties.

### Time-lapse BLI of clock gene expression in primary astrocytes using ELuc

**Figure 2:**
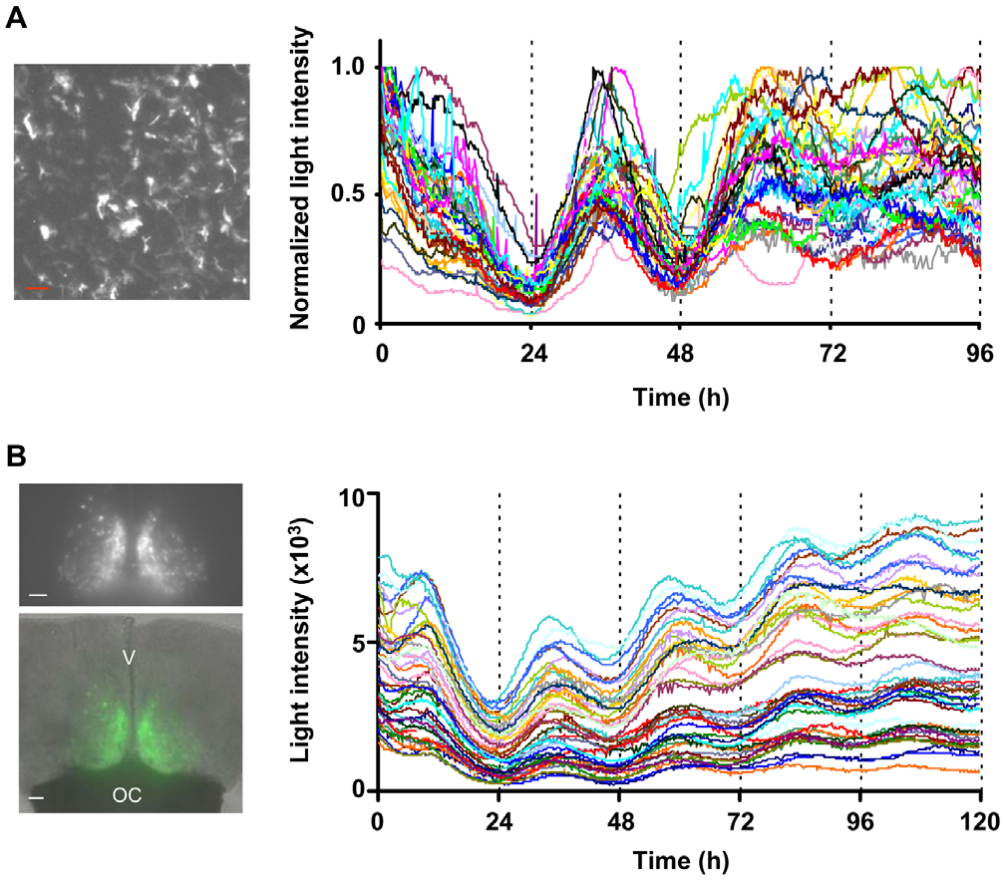
Long-term single cell imaging of transcriptional oscillation in living cells using ELuc. (**A**) Representative CCD image of *mPer2* promoter-driven ELuc luminescence in rat primary astrocytes (scale bar, 100 µm) (left panel) and recordings of luminescence from 40 individual cells (right panel). Images were acquired using 9 min of exposure time at intervals of 10 min with a 4× objective lens (numerical aperture (NA), 0.5). Signals were normalized to maximum count. (**B**) CCD image of *mBmal1* promoter-driven ELuc luminescence from SCN slices of *Bmal1*::ELuc transgenic mice (Bmal1-ELuc^A4^, upper left panel) and merged photograph of bright-field and luminescence images (green) (lower left panel). Images were taken using 10 min exposures at intervals of 15 min and a 4× objective lens (scale bars, 100 µm). V and OC are third ventral and optic chiasm, respectively. Recordings of luminescence from 44 individual cells were plotted (right panel).

Next, we performed single-cell time-lapse BLI of ELuc luminescence driven by the *mPer2* promoter in rat primary astrocytes, as a bright luminescent probe is required to capture the emission from primary cultured cells at single-cell resolution because of the low transfection efficiency inherent to this type of culture. The reporter plasmid mPer2-ELuc-PEST was transiently transfected into cultured astrocytes by electroporation and ELuc luminescence after stimulation with dexamethasone was recorded using 9 min of exposure time at 10 min intervals for 96 h on a luminescence microscope (CellGraph, ATTO) (Figure 2A). Using a photomultiplier to record luminescence signals, we noted that ELuc expressed in primary cultures emitted a signal that was 16-fold higher than that of FLuc (Figure S4A), which was similar to the measurements obtained in NIH3T3 cells (Figure 1A). As shown in Figure 2A (and Movie S1), we obtained a significantly bright signal that allowed the quantification of gene expression. Expression profiling in individual cells (n = 40) revealed that almost all cells showed circadian oscillation of *mPer2*-driven ELuc luminescence, without remarkable damping, however, this oscillation progressively drifted out of phase after three cycles. This finding may be attributed to the loss of synchrony among cells (Figure S4C), similar to that observed in cultured fibroblasts [3], [17].

### Time-lapse BLI of clock gene expression in the suprachiasmatic nucleus (SCN) using ELuc

To verify whether ELuc can be used at the single-cell level to quantify gene expression in tissues, we generated transgenic mice that expressed ELuc under the control of the *mBmal1* clock gene promoter [18]. Among the 10 transgenic lines obtained, we chose the Bmal1-ELuc^A4^ line because it exhibited the highest light output in several tissues (data not shown). ELuc luminescence from slice cultures of the SCN, which functions as a central clock and is located in the hypothalamus, was imaged using a luminescence microscope. Luminescence was recorded using 10 min of exposure time at 15 min intervals for 120 h without binning. We successfully detected a significantly bright signal that allowed the quantification of gene expression in individual cells, even at the 10 min exposure (Figure 2B and Movie S2). The 44 individual cells quantified in this way exhibited robust circadian oscillation of the *Bmal1*-driven-ELuc luminescence, with a tightly regulated period (Figure S5). An exposure of 30–60 min is generally needed for the recording of *mPer1*- or *mPer2*-driven-FLuc luminescence from transgenic mouse SCN slices [19]–[21]. Therefore, our result demonstrates that ELuc allows single-cell imaging analysis of tissue cultures with a much higher temporal resolution than that available using FLuc, although the promoter and the imaging equipment used in the two situations were different.

### Subcellular BLI using FLuc and ELuc

**Figure 3:**
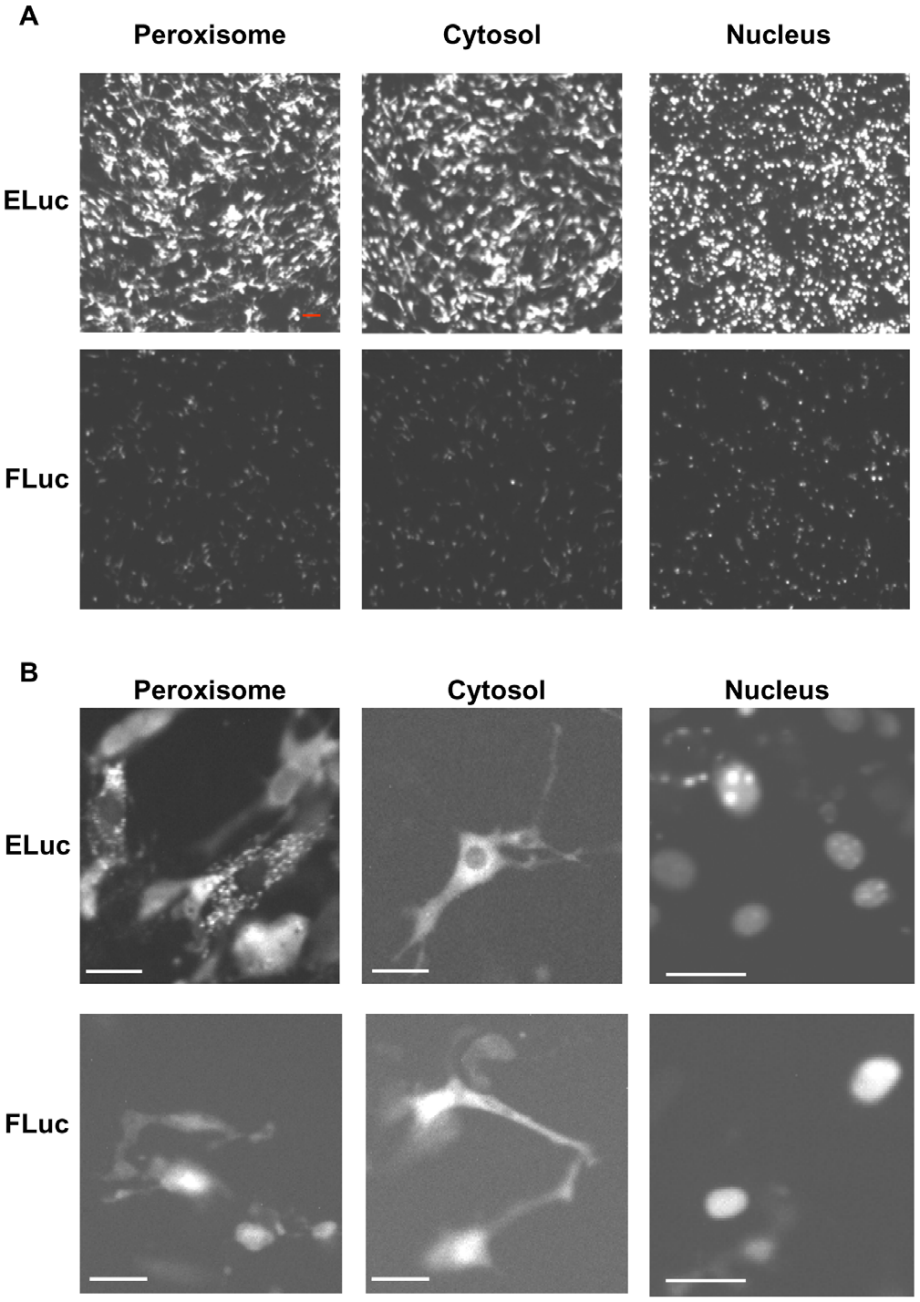
Representative luminescence CCD images of subcellular-targeted ELuc and FLuc in NIH3T3 cells. (**A**) Luminescence images of peroxisome- (left panels), cytosol- (middle panels), and nucleus- (left panels) targeted ELuc (upper panels) and FLuc (bottom panels). Expression plasmids for the subcellular-targeted expression of luciferases under the control of the CMV promoter were transiently transfected into NIH3T3 cells. Images were acquired when the signals reached the maximum using 3 min of exposure time and a 4× objective lens without binning (scale bars, 100 µm). The contrast of all images was adjusted equally. (**B**) Luminescence images of the luciferase-expressing cells shown in A, as acquired with a 40× objective lens (NA, 0.9) (scale bars, 30 µm). Images were acquired using a 3 min exposure without binning.

Subcellular BLI has been performed in luminescent dinoflagellates by capturing their endogenous luciferase emission using an image-intensifier system [22]. However, as far as we know, subcellular BLI with introduction of an exogenpus luciferase gene has never been performed. Although FLuc is used to monitor gene expression by BLI at the single-cell level, it is difficult to perform luminescence imaging at the subcellular level in mammalian cells because of the insufficiency of its light output intensity. Therefore, we next attempted to perform BLI of organelles using ELuc. Peroxisome-, cytosol- and nucleus-localized ELuc and FLuc were transiently expressed in NIH3T3 cells under the control of the CMV promoter, and their luminescence was recorded using 3 min of exposure time (Figure 3A). We found that ELuc localized to peroxisomes and nuclei, which was verified by confocal fluorescence imaging using peroxisome- and nucleus-targeted GFP (Figure S6), as reported for FLuc [23], [24]. As shown in Figure 3A, the quantification of the signal intensity from 150 individual cells revealed that ELuc produced 6-, 18-, and 15-fold higher luminescent signals in the peroxisome, cytosol, and nucleus, respectively, when compared with FLuc. Interestingly, the subcellular localization of ELuc was clearly imaged when images were captured using a 40× objective lens (Figure 3B). Notably, peroxisome-targeted ELuc exhibited a typical peroxisomal dot-like pattern. In contrast, the subcellular localization images for FLuc were less clear (i.e., low resolution), even though FLuc allows nuclear imaging.

To investigate the differences in the resolution of subcellular imaging when ELuc and FLuc are used as probes, we examined the relationships between the D-luciferin concentration and the light intensities of the luciferases in living cells (Figure S7A). The cytosol-targeting expression vector pCMV–Flag::FLuc or pCMV–Flag::ELuc(cyto) was transiently transfected into NIH3T3 cells, and the luminescence was measured for 72 h in real-time with a luminometer (Kronos). We noted that each luciferases-expressing cell displayed the same kinetic pattern at all the luciferin concentrations tested, although the ELuc-expressing cells showed slower increase and decay profiles compared with those of FLuc, similar to the measurements made *in vitro* (data not shown). The inset of Figure S7A shows the dose–response of the D-luciferin concentrations against the peak luminescence intensities of the luciferases-expressing cells, in which each value was normalized to the count at 2000 µM luciferin. The light intensities of both luciferases increased with increasing D-luciferin concentrations in the same dose-dependent manner, and reached a plateau at 1000 µM. However, the light output of the FLuc-expressing cells was markedly lower than that of ELuc-expressing cells at all the luciferin concentration tested (Figure S7A). We next examined whether high-resolution CCD images of subcellular-localized FLuc could be captured. Imaging was performed at the highest luciferin concentration (2000 µM), which was 10-fold higher than the concentration used for the measurements shown in Figure 3. However, the images were still unclear even at that luciferin concentration (Figure S7B), indicating that the lower-resolution images of FLuc were simply the result of its insufficient luminescence in living cells. We noted that the subcellular images were also not sharp, even when taken using longer exposure times (data not shown). Thus, although FLuc is used for BLI at the single-cell level, the light intensity produced is inadequate for subcellular imaging, as performed under our experimental conditions. These results appear to indicate that ELuc allows the live imaging of changes in intracellular localization of proteins fused to this molecule.

### Time-lapse BLI of the intracellular trafficking of importin α using ELuc

**Figure 4:**
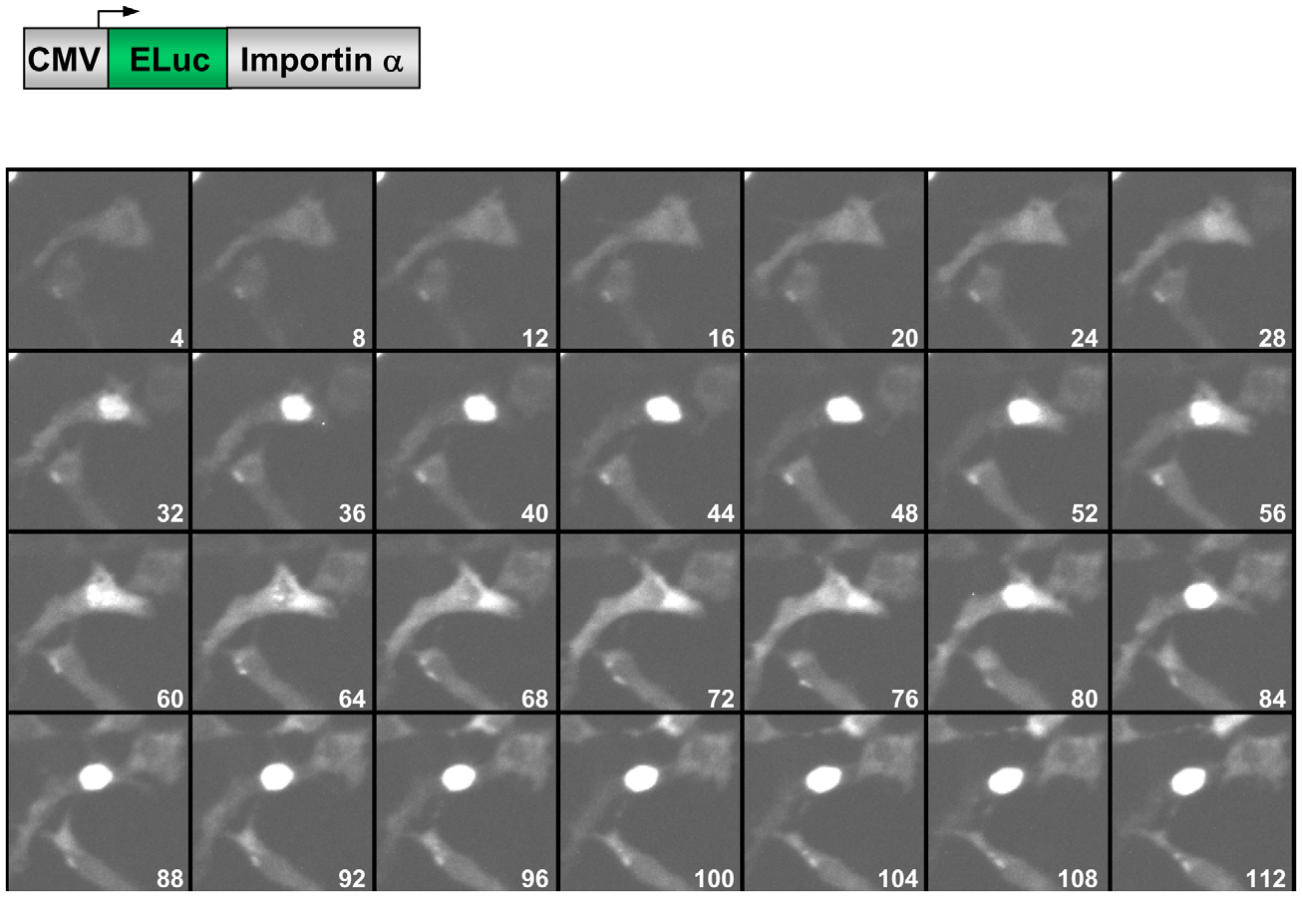
Time-lapse luminescence imaging of the nucleocytoplasmic shuttling of ELuc::importin α in NIH3T3 cells. The expression plasmid carrying ELuc::importin α was transiently transfected into NIH3T3 cells. Images were acquired after 3 h of transfection using 3 min of exposure time at intervals of 4 min with a 40× objective lens without binning. Numbers indicate minutes. A schematic drawing of the expression plasmid is shown on the upper panel.

To explore the possibility of performing time-lapse BLI of intracellular trafficking of proteins using ELuc, we attempted to image the trafficking pattern of mouse importin α (mRch1) [25], [26], which is a representative nucleocytoplasmic shuttling protein. We removed the peroxisome targeting signal located at the extreme C-terminus of ELuc and fused importin α at this C-terminus. The fusion construct was transiently expressed in NIH3T3 cells under the control of the CMV promoter. Immunoblot analysis demonstrated that the fusion protein was expressed in these cells at the expected molecular size of 120 kDa (Figure S8). Time-lapse BLI was recorded 3 h after transfection using 3 min of exposure time at 4 min intervals (Figure 4 and Movie S3). The luminescence signal was initially detected in the cytosol at 480 min after commencement of measurements (time = 0); the signal increased gradually in the nucleus after 28 min, accompanied by a decrease of the signal in the cytosol. Conversely, the signal in the nucleus gradually decreased after 44 min, with a concomitant increase in cytosolic signal, up to 76 min. Accumulation of the signal in the nucleus was again observed from 80 to 112 min. In previous studies, time-lapse fluorescence imaging using GFP-fused importin α demonstrated that this protein reversibly migrates into the nucleus from the cytosol after treatment with UV irradiation in a time-dependent manner [27], almost identical to what was observed in the present study. This demonstrates the accuracy of intracellular BLI using ELuc as a probe.

When we performed continuous time-lapse imaging for up to 20 h, the ELuc::improtin α protein was completely retained within the nucleus after two cycles of shuttling (data not shown). It has been reported that the nuclear accumulation of importin α induced by cellular stress is triggered by the collapse of the gradient of Ran, a small GTPase that is involved in the nuclear cytoplasm shuttling of importin α, resulting in the rapid nuclear accumulation importin α with impairment of its nuclear export [27]. Therefore, it is reasonable to assume that the nuclear retention of the ELuc::improtin α protein here was caused by the collapse of the Ran gradient, although we can not correlate the nucleocytoplasmic shuttling observed under our experimental conditions and the ambient stress condition. It is also noted that the repeated nuclear migration of ELuc::improtin α protein shown in Figure 4 was observed in approximately 10% of luminescent cells, whereas this protein was continuously retained in the nucleus in the other 90% of cells. This may reflect the excessive expression levels of the fusion protein, as a moderate expression level is required for retention of the protein in the cytosol [27].

Thus, we successfully used ELuc to visualize the nucleocytoplasmic shuttling of importin α at a periodicity of approximately 30 min. This represents the first reported observation of time-lapse imaging of the intracellular movement of a protein by means of BLI.

### Conclusions

In conclusion, we have developed ELuc, which is a luciferase with enhanced brightness compared with FLuc. This allowed us to successfully image not only longitudinal gene expression in primary cells and tissues with high temporal resolution, but also the intracellular trafficking of a nucleocytoplasmic shuttling protein with high spatial resolution. Therefore, ELuc is greatly advantageous for the achievement of high spatiotemporal definition BLI for the continuous visualizing gene expression and trafficking of proteins at the single-cell and subcellular levels. Furthermore, in combination with red- and/or orange-emitting luciferases [13], [28], the system developed in this study can be applied to multicolor BLI, thereby enabling the simultaneous analysis of multiple cellular events.

## Materials and Methods

### Optimization of the *P. termitilluminans* luciferase sequence for mammalian expression

To optimize the codons of *P. termitilluminans* luciferase [5] (GeneBank accession number AF116843) for mammalian expression, codon usage data were obtained from the *Mus musculus* database of the Kazusa DNA Research Institute (http://www.kazusa.or.jp/codon/cgi-bin/showcodon.cgi?species=10090). To delete putative transcription factor binding sites within the cDNA sequence, the sites were identified and deleted using MatInspector sequence analysis software (Genomatix, Munich, Germany). All nucleotide substitutions were designed without changing the deduced amino acid sequence, with the exception of the duplicated first methionine, which was deleted. The designed 1,629 bp double-strand cDNA was synthesized using a customized DNA synthesis service at TOYOBO (Osaka, Japan). To generate pBlue-ELuc, the cDNA was ligated to the HindIII/XbaI site of pBluescript KS(+) (Stratagene, La Jolla, CA).

### Plasmid construction

To construct expression plasmids carrying Flag-tagged wild-type luciferase and ELuc, cDNA sequences in which the start codon was replaced by an *Eco*RV site were amplified by polymerase chain reaction (PCR) using pB1_py311_ [5] and pBlue-ELuc, respectively, as the templates. The amplified products were ligated into the EcoRV/KpnI (for wild-type luciferase) and EcoRV/XhoI (for ELuc) sites of pCMV-Tag2B (Stratagene) downstream of the immediate CMV promoter, which resulted in the pCMV-Flag::PTLuc and pCMV-Flag::ELuc constructs. pSV40-ELuc was generated by replacing the *Nco*I and *Xba*I fragment of the FLuc expression vector pGVC2 (Toyo Ink, Tokyo, Japan) with ELuc excised from the pBlue-ELuc plasmid. To generate destabilized luciferases in which the PEST element of the mouse ornithine decarboxylase was fused in-frame to the C-terminus of ELuc (dELuc) and FLuc (dFLuc), the PEST sequence (in which the *Nco*I site was deleted without changing the deduced amino acid sequence) was PCR-amplified using pd1EGFP-N1 (Clontech, Palo Alto, CA) as a template. To generate pGEM-PEST, the amplified fragment was subcloned into pGEM-TEasy (Promega, Madison, WI). ELuc and FLuc in which the stop codon was replaced by an *Eco*RV site were PCR-amplified using pBlue-ELuc and the pGVC2 as templates, respectively. The amplified fragments were ligated into the NcoI/EcoRV site of pGEM–PEST. To generate the pSV40–dELuc and pSV40–dFLuc constructs, PEST-fused ELuc (dELuc) and FLuc (dFLuc) were excised using *Nco*I and *Xba*I and ligated into the NcoI/XbaI site of pGVC2, in which the FLuc was removed.

To construct reporter vectors carrying the *mPer2* promoter, the *mPer2* promoter fragment (−279 to +112 bp, where +1 indicates the putative transcription start site) was PCR-amplified from C57BL/6J mouse genomic, and cloned into the NheI/XhoI site of pGL3–Basic (Promega). The FLuc was replaced with the *Nco*I and *Xba*I fragments of pSV40–dELuc and pSV40–dFLuc, resulting in mPer2-dELuc and mPer2-dFLuc, respectively.

To generate the cytosol-targeting ELuc expression vector pCMV-Flag::ELuc (cyto), the ELuc cDNA in which the peroxisome-targeting signal (PTS; Ser-Lys-Lue) at the extreme C-terminus was deleted by PCR using pBlue-ELuc as the template, and the product was ligated to the EcoRV/XhoI site of pCMV-Tag2B. To construct the peroxisome-targeting FLuc expression vector pCMV-Flag::FLuc (pox), the FLuc cDNA in which a PTS was introduced at the extreme C-terminus was PCR-amplified using pGVC2 as the template, and the product was ligated to the EcoRV/XhoI site of pCMV-Tag2B. To construct expression plasmids carrying nucleus-targeting ELuc and FLuc, the cDNA sequences in which the PTS of ELuc and the STOP codons of both luciferases were replaced by a *Not*I site, and were PCR-amplified using pBlue-ELuc and pGVC2 as templates, respectively. To obtain the pCMV-Myc::ELuc (nuc) and pCMV-Myc::FLuc (nuc) constructs, the amplified fragments were ligated into the NocI/NotI site of pCMV/myc/nuc (Invitrogen, Carlsbad, CA) in which a triple nuclear localization signal (NLS) from SV40 large T antigen was introduced downstream of the multiple cloning site, for C-terminal fusion to the luciferases.

For confocal fluorescence imaging analysis, we generated a peroxisome-targeting EGFP in which a PTS was introduced at the extreme C-terminus of EGFP. The EGFP cDNA was PCR-amplified using EGFP-N1 (Clontech) as a template, and the product was cloned into pcDNA3.1/V5-His TOPO (Invitrogen) to yield pCMV- EGFP (pox).

To construct an expression plasmid carrying ELuc::importin α, the ELuc cDNA in which the PTS and the STOP codon were replaced by an *Eco*RV site was PCR-amplified using pBlue-ELuc as a template, and the product was ligated into the HindIII/EcoRV site of pcDNA3 (Invitrogen). The importin α-coding sequence (mRch1, GeneBank accession number D55720) in which the start codon was replaced by an *Eco*RV site was PCR-amplified using pGEX-2T-PTAC58 [25] (kind gift from Dr. Y. Yoneda of Osaka University) as a template, and the product was ligated to the *Eco*RV and *Xho*I sites downstream of ELuc to yield pCMV-ELuc:: importin α. All constructs were verified by sequencing.

### Cell culture

Mouse NIH3T3 cells (RCB1862) were grown in Dulbecco's Modified Eagle medium (DMEM, Sigma-Aldrich, St. Louis, MO) supplemented with 10% fetal bovine serum (FBS, ICN Biochemicals, Aurora, OH) in a humidified atmosphere containing 5% CO_2_ at 37°C. To prepare rat primary astrocytes, cerebral cortices were cut into small pieces, incubated in papain solution at 37°C for 15 min, and then separated by gentle trituration passes. Cells were plated onto a flask and cultured in DMEM supplemented with 10% FBS at 5% CO_2_ for 3–4 h and the cultures were washed with phosphate buffered-saline twice. The dissociated cells were cultured in DMEM supplemented with 10% FBS for over 2 weeks. Confluent cells were harvested and placed in a new flask after a two-fold dilution.

### Mice and preparation of SCN slices

The reporter plasmid mBmal1 915-ELuc, which carried the 5′ flanking region (−816 to +99 bp) of the *mBmal1* promoter, was constructed by replacing the NcoI/XbaI fragment of the Bp/915-Luc vector [18] (a kind gift from Dr. M. Ikeda of Saitama Medical University) with ELuc. Transgenic mice were generated on the C57BL/6J background as described elsewhere. Among the 10 transgenic lines obtained, we used the Bmal1^ELuc-A4^ line because it showed the highest light intensity of the various tissues examined. The mice were kept in the heterozygous state and maintained in LD12∶12. Six-month-old mice were decapitated and the brain was removed. Coronal sections of the brain (250 µm thickness) were prepared using a microslicer (Dosaka, Osaka, Japan) and were transferred to ice-cold Hank's balanced salt solution supplemented with 10 mM Hepes/NaOH (pH7.0), 1.76 mg/ml NaHCO_3_, 97 U/ml penicillin, and 100 µg/ml streptomycin. Bilateral SCNs connected with optic chiasms were trimmed to approximately 2 mm squares. The procedure was strictly in accordance with the protocols approved by the Institutional Animal Care and Use Committee of the National Institute of Advanced Industrial Science and Technology.

### Real-time measurement of bioluminescence using a luminometer

NIH3T3 cells were seeded in 35 mm dishes one day before transfection. One hundred nanograms of mPer2-dELuc or mPer2-dFLuc were cotransfected into NIH3T3 cells with 100 ng of the expression plasmid pCMV-CLuc [29] carrying secreted luciferase from *Cypridina noctiluca* (CLuc) and 1 µg of pBluescript SK(−). Rat primary astrocytes were cultured and 2×10^6^ cells were suspended in 100 µl of rat astrocyte Nucleofactor (Amaxa, Köln, Germany), which was followed by the cotransfection of 4 µg of mPer2-dELuc or mPer2-dFLuc with 1 µg of pCMV-CLuc using electroporation and the program T-20 of the Nucleofactor electroporator (Amaxa). Transfected cells were seeded on 35 mm dishes. Transfected NIH3T3 cells and primary astrocytes were cultured for 1–2 days until confluence, and an aliquot of the culture medium containing secreted CLuc was collected. CLuc activity was measured using the CLuc reporter assay system (ATTO, Tokyo, Japan), according to manufacturer's instructions. Transfected cells were treated with 100 nM dexamethasone (Nacalai Tesque, Kyoto, Japan) for 2 h and the medium was replaced with DMEM without phenol red (Gibco-BRL, Grand Island, NY) supplemented with 10% FBS and 200 µM D-luciferin (TOYOBO) and overlaid with mineral oil (Sigma-Aldrich) to prevent evaporation. Bioluminescence was recorded for 1 min at intervals of 19 min under a 5% CO_2_ atmosphere at 37°C using the dish-type luminometer AB2500 Kronos (ATTO).

### CCD imaging of bioluminescence

Transfected NIH3T3 cells or primary astrocytes were grown on 35 mm glass-bottom dishes (Iwaki, Tokyo, Japan) until confluence, and an aliquot of medium was collected to measure CLuc activity. For BLI of subcellular-localized luciferases, 2 µg of expression plasmids were cotransfected with 100 ng of pCMV-CLuc into NIH3T3 cells. For imaging of *mPer*2-driven luciferase luminescence, cells were treated with 100 nM dexamethasone for 2 h before measurement. The culture medium was replaced with DMEM without phenol red and supplemented with 10% FBS, 25 mM Hepes/NaOH (pH7.0), and 200 µM D-luciferin, and was overlaid with 2 ml of mineral oil. For imaging of ELuc BLI in SCN slices, the slices were placed on a culture membrane (Millicell-CM, Millipore, Billerica, MA) with 1.3 ml of DMEM without phenol red supplemented with 10 mM Hepes/NaOH (pH7.0), B27 supplement (Invitrogen), and 200 µM D-luciferin, and the dish was sealed with parafilm. BLI was performed using the luminescence microscope CellGraph (ATTO) at 37°C. CCD images were acquired using a 4× (NA, 0.5; ATTO) or 40× (NA, 0.9; Nikon, Tokyo, Japan) objective lens at 1×1 binning of the 512×512 pixel array. The luminescence intensity was quantified using Metamorph (Universal Imaging, Brandywine, PA).

### Measurement of luciferase activity in cell extracts

NIH3T3 cells were seeded one day before transfection in 24-well plates at a density of 5×10^4^ cells per well. One hundred nanograms of expression plasmid and 10 ng of the *Renilla* luciferase expression vector phRL-TK (Promega) were cotransfected using Lipofectamine PLUS (Invitrogen) according to the manufacturer's instructions. Two days after transfection, cells were lysed with 200 µl of Passive Lysis Buffer, unless otherwise noted. The ELuc and FLuc activities were measured by mixing 50 µl of cell lysate with 50 µl of Luciferase Assay Reagent II (Promega) or PicaGene (Toyo Ink) containing D-luciferin as a substrate for 20 s using an AB-2250 luminometer (ATTO). *Renilla* luciferase activity was measured separately for 20 s by mixing 50 µl of lysate and 50 µl of 200 µM coelenterazine (Sigma-Aldrich) dissolved in 10 mM Tris/HCl (pH 7.4). The activities of wild-type *P. termitilluminans* luciferase, ELuc, and FLuc were normalized to the *Renilla* luciferase activity.

### Spectrum measurement

To measure the bioluminescent spectrum in cell extracts and in live cells, 400 ng or 2 µg of expression plasmid was transfected into NIH3T3 cells that were seeded in a 24-well plate or in a 35 mm dish, respectively, and cultured for 2 days. Cells in the 24-well plate were lysed in 200 µl of Passive Lysis Buffer and the spectrum was measured by mixing 15 µl of Luciferase Assay Reagent II into 15 µl of the cell extract. For measurement of the spectrum in live cells, the culture medium of cells grown in a 35 mm dish was replaced with DMEM without phenol red supplemented with 10% FBS and 200 µM D-luciferin, and the dish was placed on the sample stage of a spectrophotometer. Spectrum measurement was carried out using an AB-1850 spectrophotometer (ATTO) for 1 min with 1 mm slit width. All spectra were corrected for the spectral sensitivity of the equipment and normalized.

### Expression and purification of recombinant luciferase

To generate the plasmids for the expression of the recombinant proteins in *Escherihia coli*, the FLuc and ELuc cDNAs were subcloned into the *Xho*I/*Xba*I and *Hind*III/*Xba*I sites, respectively, of the pCold II vector (Takara Bio, Kyoto, Japan), so that the hexahistidine tag was fused in-frame to the N-terminus of the luciferase. The resulting plasmid was transformed into *E. coli* BL21 (DE3), and grown in Luria–Bertani medium. Luciferase expression was induced at 15°C for 24 h in the presence of 0.1 mM isopropyl-β-D-thiogalactopyranoside. The harvested cells were resuspended in buffer A (50 mM sodium phosphate (pH 7.0), 300 mM sodium chloride) containing 10 mM imidazole, sonicated, and centrifuged. The histidine-tagged luciferase was then bound to Ni–NTA agarose beads (Qiagen, Valencia, CA), washed twice with buffer A containing 20 mM imidazole, and eluted with buffer A containing 250 mM imidazole. Glycerol was added to the eluates to a final concentration of 8% (w/v). SDS–PAGE was used to confirm the amounts and purity of the recombinant luciferases.

### Activity and kinetics measurements of the purified luciferase

The activities and temporal kinetics of the luciferases were measured with an AB-2250 luminometer (ATTO) after 0.1 µg of purified luciferase was mixed with 100 µl of PicaGene for 60 s. The decay kinetics were measured by mixing 10 µl of 0.1 µg/ml purified luciferase solution that was diluted in 25 mM Hepes/NaOH (pH 7.0) with 90 µl of luciferin solution containing 0.4 mM D-luciferin potassium salt (TOYOBO), 3 mM ATP disodium salt, and 8 mM magnesium sulfate (Wako) using an auto injector. Luminescence was measured for 20 s at 0.2 s intervals using a microplate-type luminometer (AB2350 Phelios, ATTO). All measurements were made at room temperature.

### Immunoblot analysis

NIH3T3 cells were seeded in 6-well plates at a density of 1×10^6^ cells per well one day before transfection. Two micrograms of expression plasmid was transfected into cells, incubated for 2 days, and the cells were then lysed using the M-PER extraction reagent (Pierce Biotechnology, Rockford, IL). SDS-urea-PAGE and blotting were carried out as described previously [30]. We used mouse anti-Flag M2 (Sigma-Aldrich) and mouse anti-α-tubulin (Sigma-Aldrich) as primary antibodies. The rabbit anti-ELuc polyclonal antibody was raised against purified recombinant ELuc. We used horseradish peroxidase-conjugated anti-mouse IgG (BioRad, Hercules, CA) and anti-rabbit IgG (Jackson ImmmunoResearch, West Grove, PA) as secondary antibodies. Antibodies were diluted in Can Get Signal solution (TOYOBO). Immunoreacted bands were detected using the ECL plus kit (GE Healthcare, Freiburg, Germany) according to the manufacturer's instructions.

### Measurement of the stability of luciferases in NIH3T3 cells

To estimate the functional half-life of ELuc and FLuc in NIH3T3 cells, we used destabilized luciferases (dELuc and dFLuc) fused to the PEST element, as we could not precisely estimate the half-life of ELuc (>8 h) because of its high stability in cells (data not shown). Stability measurement was performed as described previously [31]. One hundred nanograms of the expression plasmids pSV40-dELuc and pSV40-dFLuc were independently transfected into NIH3T3 cells seeded in 48-well plates. One day after transfection, the culture medium was replaced with DMEM supplemented with 10% FBS and 100 µM cycloheximide and incubated for 20 min, to block protein synthesis. After 20 min (time = 0), the incubation was continued in the same medium. At the indicated times, cells were lysed with 200 µl of the Passive Lysis Buffer and luciferase activity was measured by mixing 20 µl of the cell lysate with 20 µl of the Luciferase Assay Reagent II and using an AB-2250 luminometer.

### Confocal fluorescence imaging

NIH3T3 cells seeded in 35 mm glass-bottom dishes were cotransfected with 2 µg of pCMV-Flag::ELuc(pox) and 0.5 µg of pCMV-EGFP(pox) or 2 µg of pCMV-Myc::ELuc(nuc) and 0.5 µg of pAcGFP1-Nuc (Clontech). One day after transfection, cells were fixed with 4% (w/v) paraformaldehyde and treated with 0.3% (w/v) TritonX-100. Cells were then incubated with anti-Flag M2 or anti-Myc (9E10, Santa Cruz Biotechnology, Santa Cruz, CA) antibodies and stained with Cy5-conjugated anti-mouse IgG (Santa Cruz). ELuc and GFP were visualized using laser confocal microscopy (BioRad).

## Supporting Information

Figure S1 Nucleotide sequences of P. termitilluminans wild-type luciferase and ELuc. cDNA sequences of wild-type luciferase and sequence-optimized luciferase, ELuc, are shown in the upper and lower rows, respectively. Identical sites are marked by asterisks.
(1.19 MB TIF)

Figure S2 Improvement of the expression and light intensity of ELuc in NIH3T3 cells. (A) Western blot analysis of the expression of wild-type luciferase and ELuc in NIH3T3 cells. NIH3T3 cells were transfected with expression plasmid carrying wild-type luciferase (pCMV-Flag::PTLuc) or sequence-optimized luciferase, ELuc (pCMV-Flag::ELuc) and cells were harvested and disrupted 48 h later. Both luciferases were detected using the anti-Flag M2 antibody. Tubulin was used as an internal control. The positions of molecular weight markers are indicated on the left margin of each panel. (B) Luminescence intensity of wild-type luciferase- and ELuc-expressing cell extracts. One hundred nanograms of the expression plasmids pCMV-Flag::PTLuc or pCMV-Flag::ELuc was cotransfected with 10 ng of phRL-TK into NIH3T3 cells. One day after transfection, cells were disrupted using 10 mM Tris/HCl (pH 7.4). The luminescent activities of wild-type luciferase and ELuc were measured and normalized to Renilla luciferase activity. The light intensity of normalized wild-type luciferase was set to 1. Error bars indicate the standard deviation (n = 6).
(0.69 MB TIF)

Figure S3 Comparison of the kinetics and light output from purified FLuc and ELuc. (A) Representative kinetics of light output from purified FLuc (orange line) and ELuc (green line). The kinetics was measured for 60 s by mixing purified protein (0.1 Î¼g) and PicaGene as a substrate. (B) Luminescence intensity of FLuc and ELuc. Signals were accumulated for 60 s, as shown in (A), and normalized to Î¼g of protein. Error bars indicate standard deviation (n = 6).
(0.66 MB TIF)

Figure S4 Real-time monitoring and single-cell imaging of mPer2 promoter-driven transcriptional oscillation in rat primary astrocytes. (A) Photomultiplier recording of mPer2 transcriptional oscillation in primary astrocytes expressing ELuc (green filled circles) and FLuc (orange filled circles). The reporter plasmids mPer2-dELuc or mPer2-dFLuc were cotransfected with pCMV-CLuc and cells were stimulated with 100 nM of dexamethasone. Bioluminescence was counted for 1 min at intervals of 19 min using luminometer (Kronos), and the respective luciferase activities were normalized to CLuc activity. The inset shows recordings where the peak values of the curves were set to 1. (B) Representative CCD image of mPer2 promoter-driven ELuc luminescence in primary astrocytes (scale bar, 100 Î¼m). We quantified 40 individual cells (yellow squares). (C) Recordings of luminescence from the 40 individual cells shown in B.
(1.89 MB TIF)

Figure S5 Time-lapse BLI of mBmal1 promoter-driven transcriptional oscillation in an SCN slice of Bmal1-ELuc transgenic mice. (A) Representative CCD image of mBma1 promoter-driven ELuc luminescence from SCN (scale bar, 100 Î¼m). We quantified 44 individual cells (yellow circles). (B) Serial CCD images of the SCN slice. Numbers indicate hours. (C) Recordings of luminescence from the 44 individual cells shown in A.
(1.77 MB TIF)

Figure S6 Confocal micrographs of peroxisome- and nuclear-localized ELuc in NIH3T3 cells. pCMV-Flag::ELuc(pox) and pCMV-EGFP(pox) or pCMV-Myc::ELuc(nuc) and pAcGFP1-Nuc were cotransfected into NIH3T3 cells. Twenty-four hours after transfection, cells were fixed and peroxisome-localized ELuc and nuclear-localized ELuc were detected using the anti-Flag M2 and anti-Myc antibodies, respectively.
(1.80 MB TIF)

Figure S7 Effects of increasing concentration of D-luciferin on the light output from FLuc- and ELuc-expressing live cells and luminescence CCD images of cytsol-targeted FLuc at higher D-luciferin concentration. (A) Relationships between the concentration of D-luciferin and peak intensities of FLuc-expressing (orange filled circles) and ELuc-expressing (green filled circles) NIH3T3 cells. Two micrograms of expression plasmid pCMV-Flag::FLuc or pCMV-Flag::ELuc(cyto) was transfected into NIH3T3 cells. One day after transfection, bioluminescence was measured using luminometer (Kronos), in real-time at various D-luciferin concentrations. Peak intensities at each luciferin concentration, as obtained by real-time measurement, are plotted in the figure. The inset shows the D-luciferin dose dependencies of FLuc and ELuc luminescence where the counts at 2000 Î¼M was set to 1. (B) Luminescence images of the cytosol-localized FLuc in NIH3T3 cells captured at 2000 Î¼M D-luciferin. NIH3T3 cells were transiently transfected with pCMV-Flag::FLuc(pox). Images were acquired when the signals reached the maximum, using a 3 min exposure time and 40× objective lens without binning.
(1.31 MB TIF)

Figure S8 Western blot analysis of the ELuc::importinα fusion protein in NIH3T3 cells. NIH3T3 cells transfected with pCMV-ELuc::importin a were harvested and disrupted 48 h after transfection. The ELuc::importin Î± fusion protein was detected using an anti-ELuc antibody. Tubulin was used as an internal control. The positions of molecular weight markers are indicated on the left margin of each panel.
(0.82 MB TIF)

Movie S1 Time-lapse BLI of *mPer2* promoter-driven ELuc luminescence from the rat astrocytes shown in Figure 2A and Figure S4. CCD images were acquired using 9 min of exposure time at 10 min intervals for 96 h with a 4× objective lens.
(2.59 MB AVI)

Movie S2 Time-lapse BLI of *mBmal1* promoter-driven ELuc luminescence from the SCN slice of Bmal1-ELuc transgenic mice shown in Figure 2B and Figure S5. CCD images were acquired using 10 min of exposure time at 15 min intervals for 120 h with a 4× objective lens.
(4.20 MB AVI)

Movie S3 Time-lapse intracellular BLI of the nucleocytoplasmic shuttling of ELuc::importinα in the NIH3T3 cells shown in Figure 4. CCD images were acquired using 3 min of exposure time at 4 min intervals for 112 min with a 40× objective lens.
(0.28 MB AVI)
