# Supplementary figures and images for: Enhanced Beetle Luciferase for High-Resolution Bioluminescence Imaging
Source: PLoS One. 2010 Apr 2;5(4):e10011. doi: 10.1371/journal.pone.0010011 (PMC2848861; doi:10.1371/journal.pone.0010011)

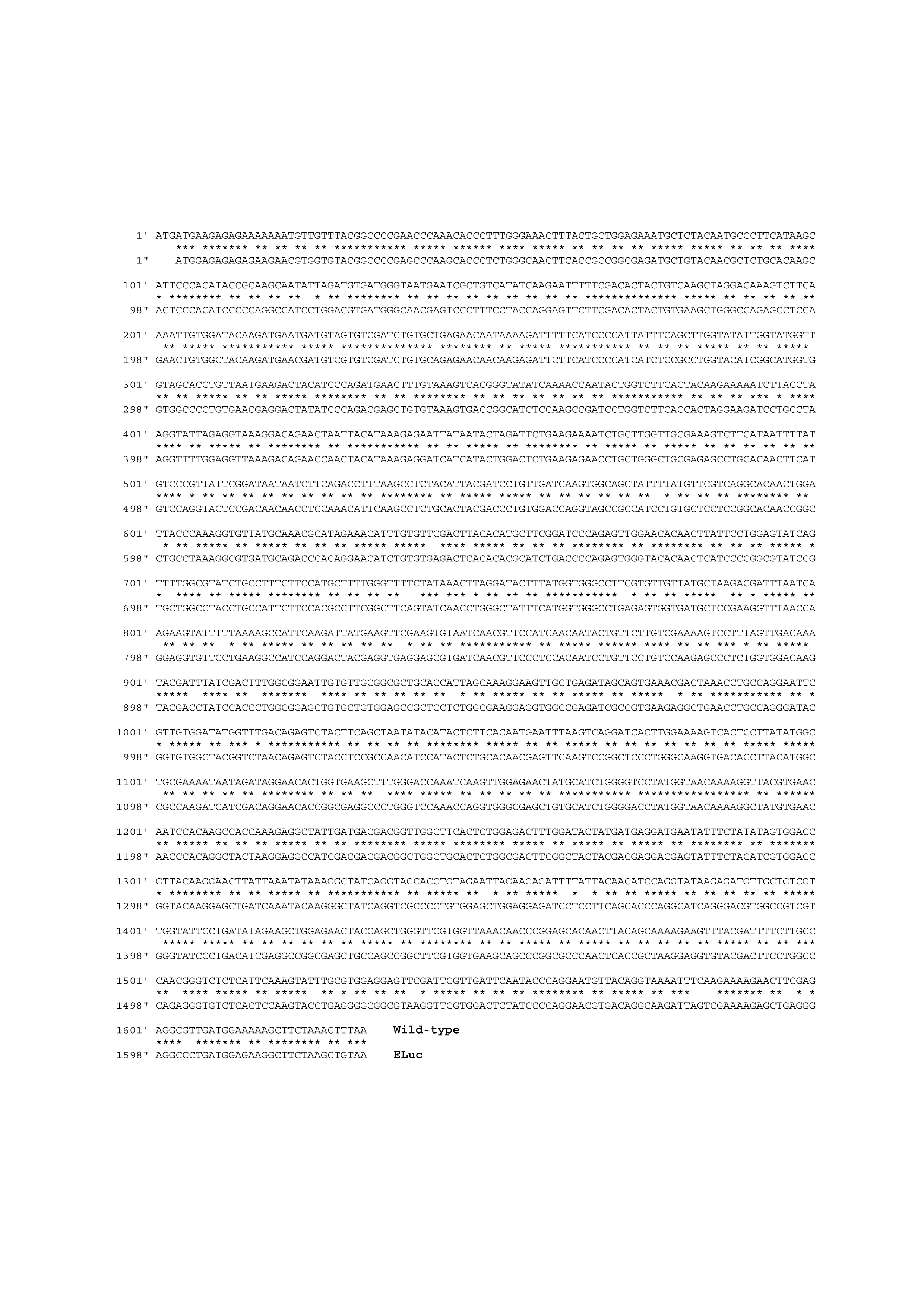

Supplement: Figure S1 — Nucleotide sequences of P. termitilluminans wild-type luciferase and ELuc. cDNA sequences of wild-type luciferase and sequence-optimized luciferase, ELuc, are shown in the upper and lower rows, respectively. Identical sites are marked by asterisks. (1.19 MB TIF) [file pone.0010011.s001.tif]

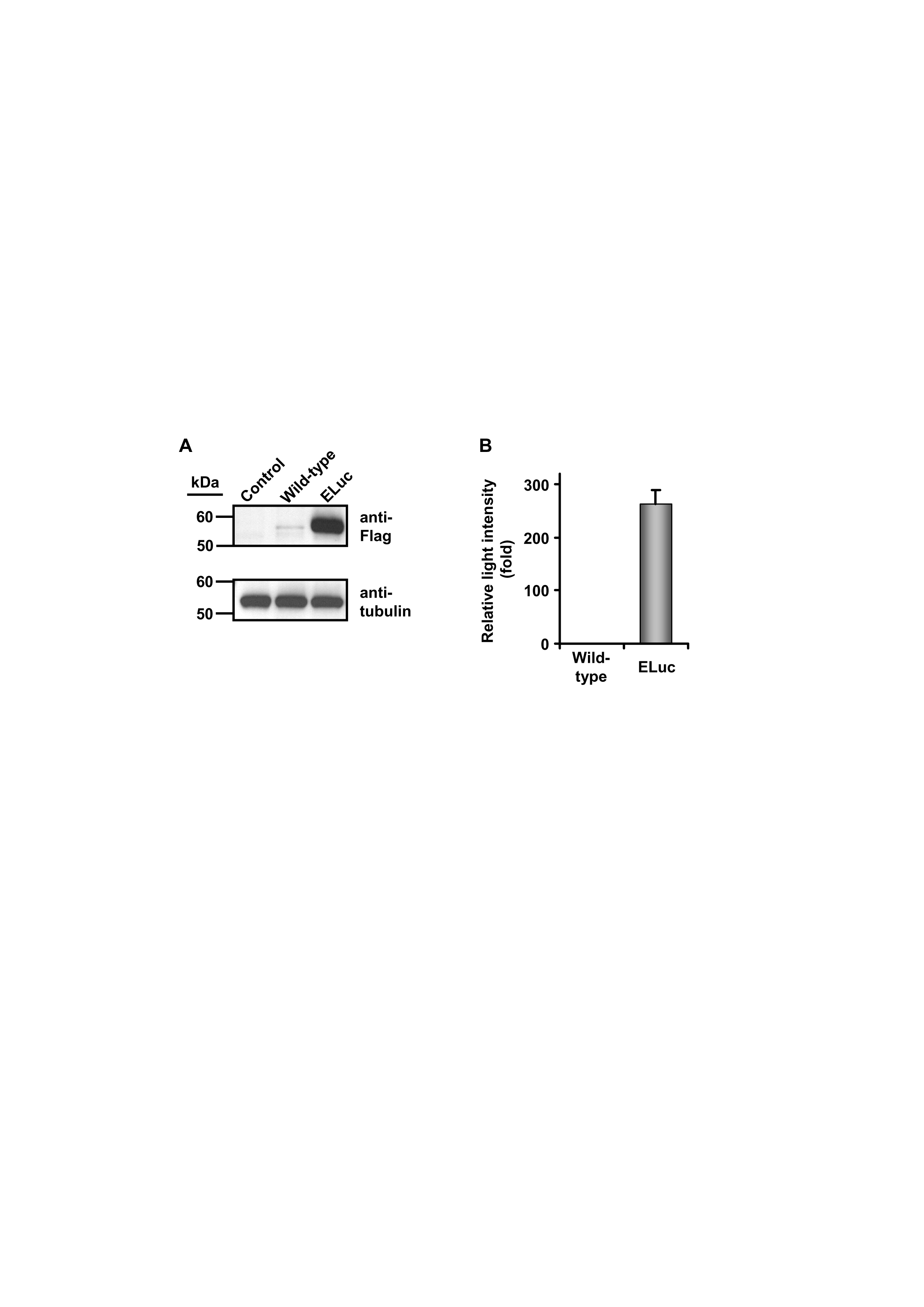

Supplement: Figure S2 — Improvement of the expression and light intensity of ELuc in NIH3T3 cells. (A) Western blot analysis of the expression of wild-type luciferase and ELuc in NIH3T3 cells. NIH3T3 cells were transfected with expression plasmid carrying wild-type luciferase (pCMV-Flag::PTLuc) or sequence-optimized luciferase, ELuc (pCMV-Flag::ELuc) and cells were harvested and disrupted 48 h later. Both luciferases were detected using the anti-Flag M2 antibody. Tubulin was used as an internal control. The positions of molecular weight markers are indicated on the left margin of each panel. (B) Luminescence intensity of wild-type luciferase- and ELuc-expressing cell extracts. One hundred nanograms of the expression plasmids pCMV-Flag::PTLuc or pCMV-Flag::ELuc was cotransfected with 10 ng of phRL-TK into NIH3T3 cells. One day after transfection, cells were disrupted using 10 mM Tris/HCl (pH 7.4). The luminescent activities of wild-type luciferase and ELuc were measured and normalized to Renilla luciferase activity. The light intensity of normalized wild-type luciferase was set to 1. Error bars indicate the standard deviation (n = 6). (0.69 MB TIF) [file pone.0010011.s002.tif]

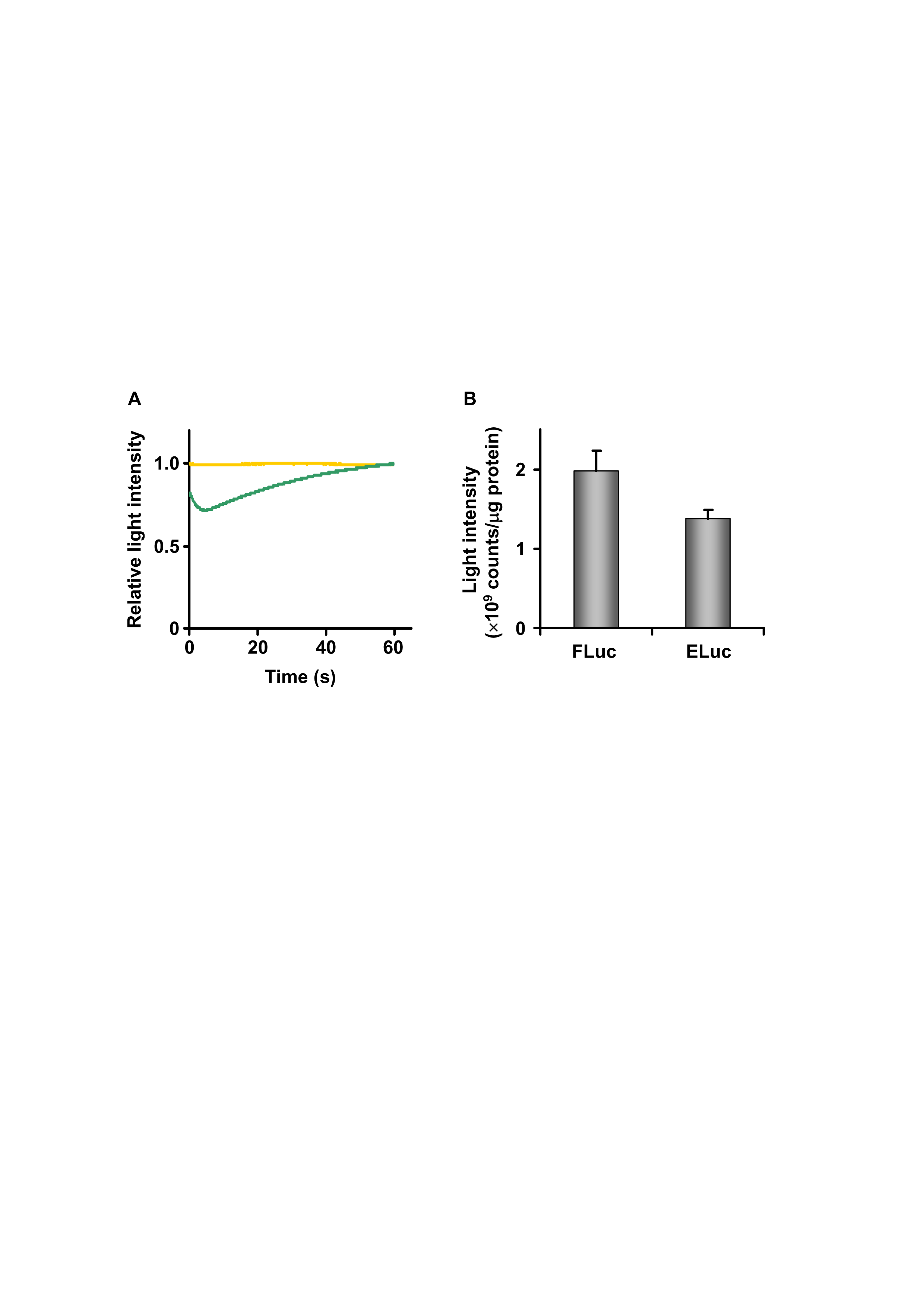

Supplement: Figure S3 — Comparison of the kinetics and light output from purified FLuc and ELuc. (A) Representative kinetics of light output from purified FLuc (orange line) and ELuc (green line). The kinetics was measured for 60 s by mixing purified protein (0.1 Î¼g) and PicaGene as a substrate. (B) Luminescence intensity of FLuc and ELuc. Signals were accumulated for 60 s, as shown in (A), and normalized to Î¼g of protein. Error bars indicate standard deviation (n = 6). (0.66 MB TIF) [file pone.0010011.s003.tif]

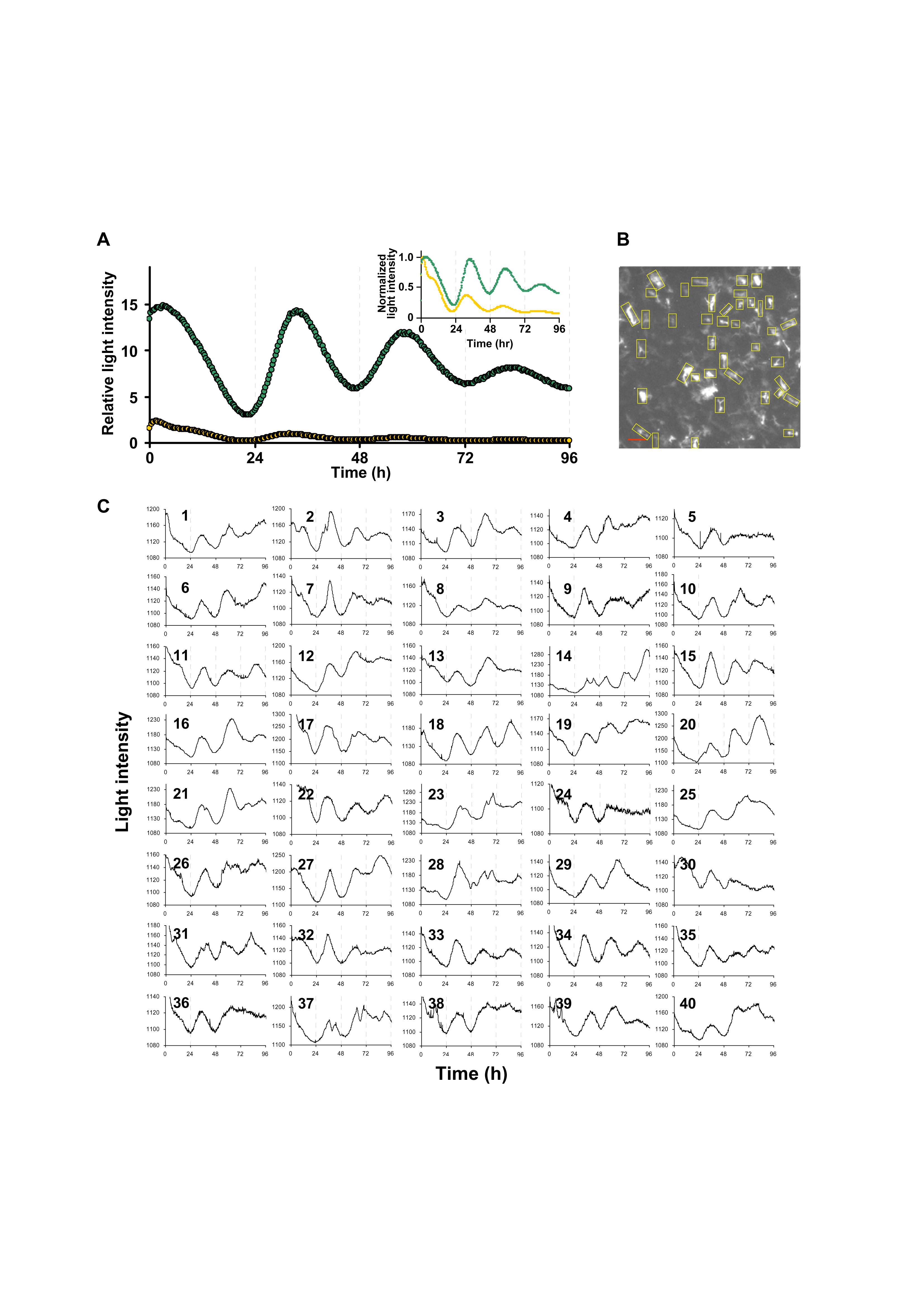

Supplement: Figure S4 — Real-time monitoring and single-cell imaging of mPer2 promoter-driven transcriptional oscillation in rat primary astrocytes. (A) Photomultiplier recording of mPer2 transcriptional oscillation in primary astrocytes expressing ELuc (green filled circles) and FLuc (orange filled circles). The reporter plasmids mPer2-dELuc or mPer2-dFLuc were cotransfected with pCMV-CLuc and cells were stimulated with 100 nM of dexamethasone. Bioluminescence was counted for 1 min at intervals of 19 min using luminometer (Kronos), and the respective luciferase activities were normalized to CLuc activity. The inset shows recordings where the peak values of the curves were set to 1. (B) Representative CCD image of mPer2 promoter-driven ELuc luminescence in primary astrocytes (scale bar, 100 Î¼m). We quantified 40 individual cells (yellow squares). (C) Recordings of luminescence from the 40 individual cells shown in B. (1.89 MB TIF) [file pone.0010011.s004.tif]

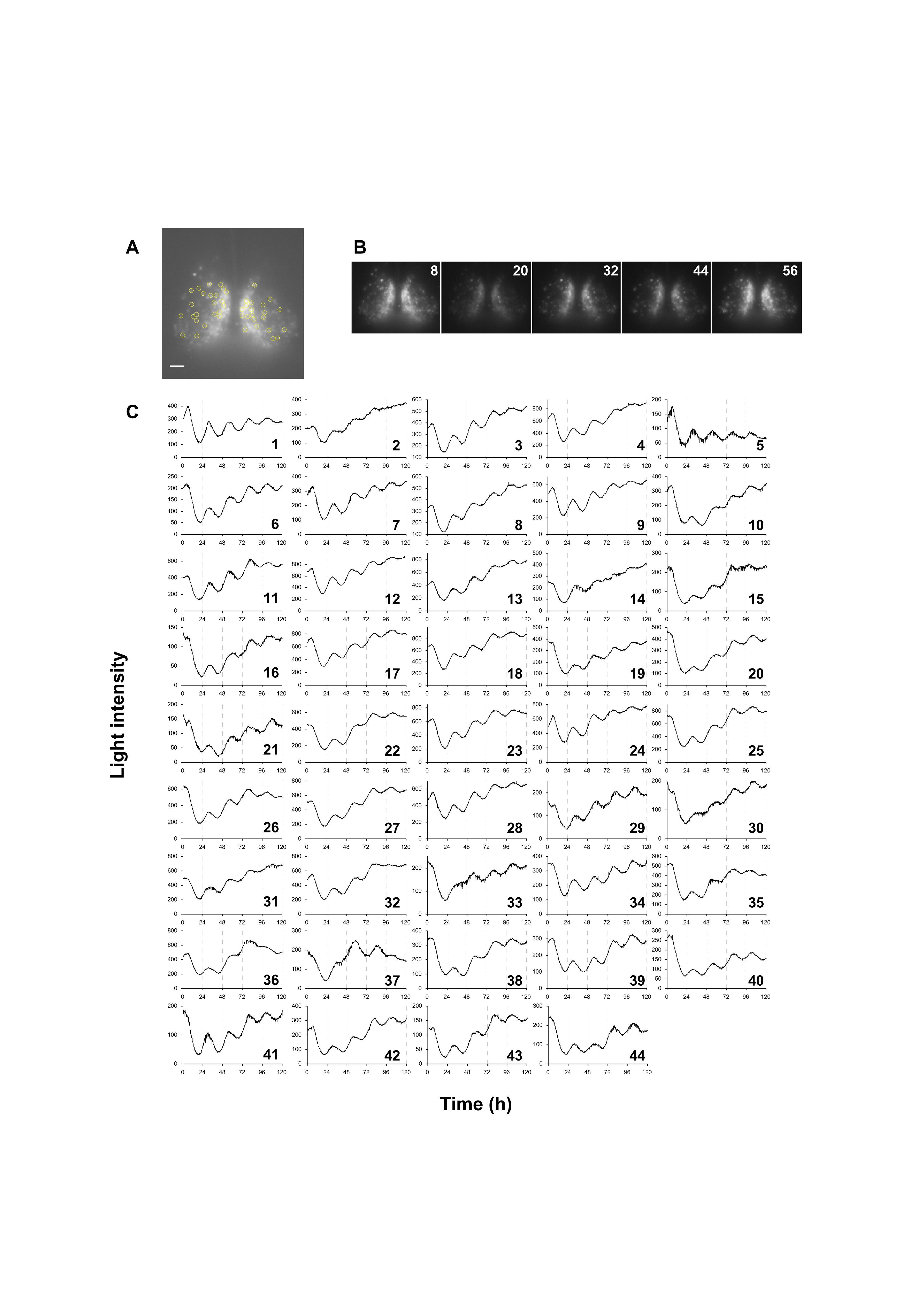

Supplement: Figure S5 — Time-lapse BLI of mBmal1 promoter-driven transcriptional oscillation in an SCN slice of Bmal1-ELuc transgenic mice. (A) Representative CCD image of mBma1 promoter-driven ELuc luminescence from SCN (scale bar, 100 Î¼m). We quantified 44 individual cells (yellow circles). (B) Serial CCD images of the SCN slice. Numbers indicate hours. (C) Recordings of luminescence from the 44 individual cells shown in A. (1.77 MB TIF) [file pone.0010011.s005.tif]

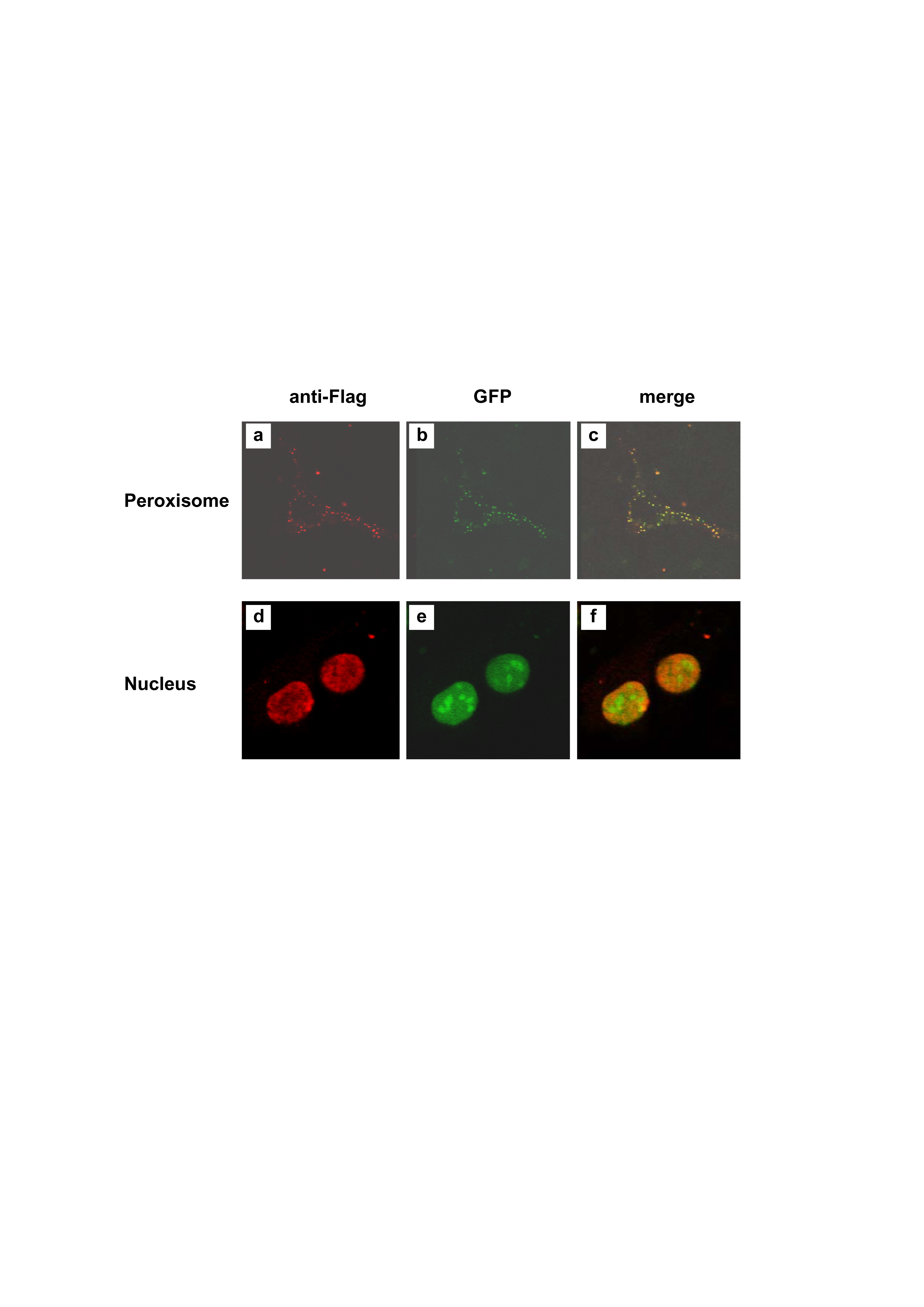

Supplement: Figure S6 — Confocal micrographs of peroxisome- and nuclear-localized ELuc in NIH3T3 cells. pCMV-Flag::ELuc(pox) and pCMV-EGFP(pox) or pCMV-Myc::ELuc(nuc) and pAcGFP1-Nuc were cotransfected into NIH3T3 cells. Twenty-four hours after transfection, cells were fixed and peroxisome-localized ELuc and nuclear-localized ELuc were detected using the anti-Flag M2 and anti-Myc antibodies, respectively. (1.80 MB TIF) [file pone.0010011.s006.tif]

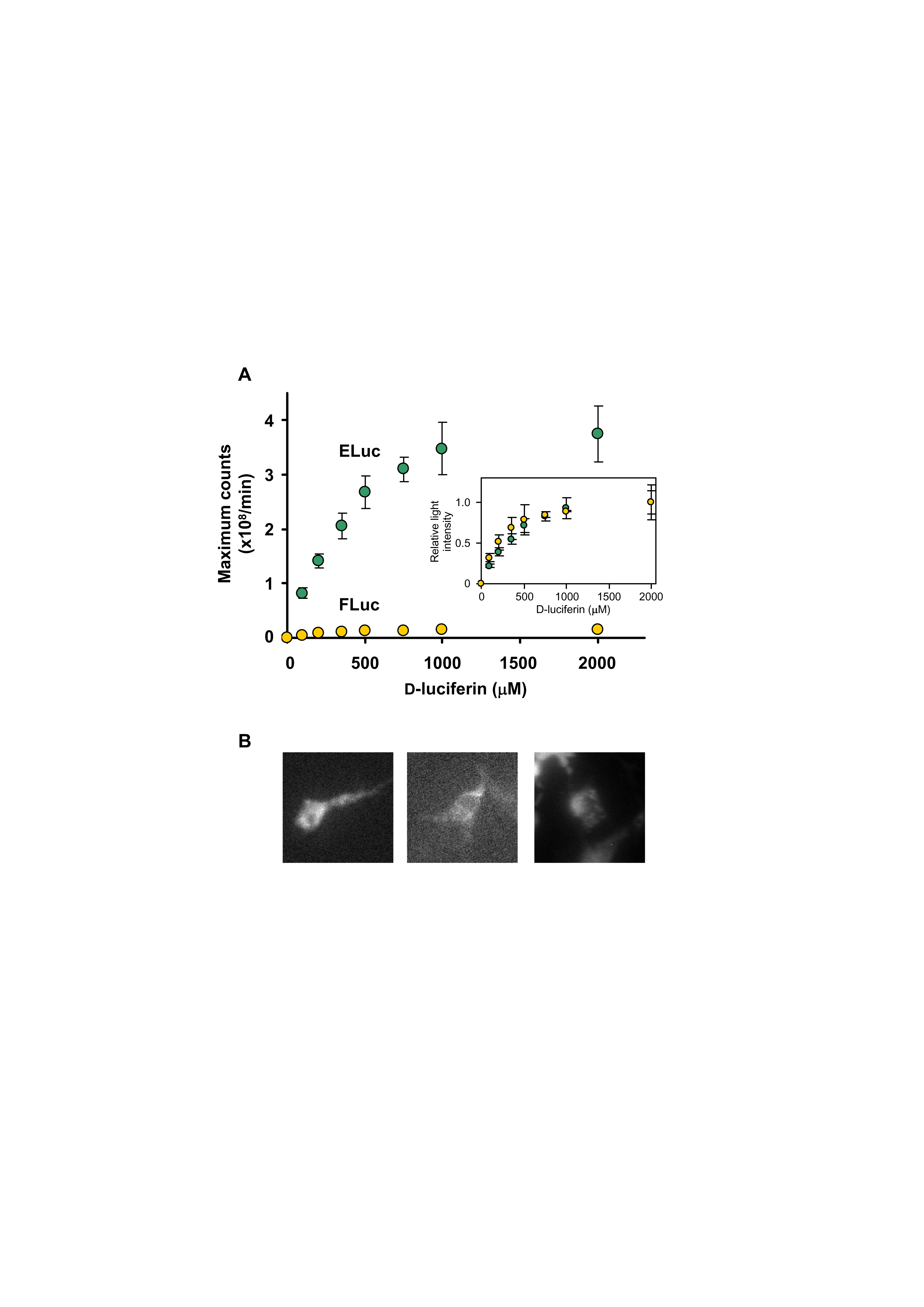

Supplement: Figure S7 — Effects of increasing concentration of D-luciferin on the light output from FLuc- and ELuc-expressing live cells and luminescence CCD images of cytsol-targeted FLuc at higher D-luciferin concentration. (A) Relationships between the concentration of D-luciferin and peak intensities of FLuc-expressing (orange filled circles) and ELuc-expressing (green filled circles) NIH3T3 cells. Two micrograms of expression plasmid pCMV-Flag::FLuc or pCMV-Flag::ELuc(cyto) was transfected into NIH3T3 cells. One day after transfection, bioluminescence was measured using luminometer (Kronos), in real-time at various D-luciferin concentrations. Peak intensities at each luciferin concentration, as obtained by real-time measurement, are plotted in the figure. The inset shows the D-luciferin dose dependencies of FLuc and ELuc luminescence where the counts at 2000 Î¼M was set to 1. (B) Luminescence images of the cytosol-localized FLuc in NIH3T3 cells captured at 2000 Î¼M D-luciferin. NIH3T3 cells were transiently transfected with pCMV-Flag::FLuc(pox). Images were acquired when the signals reached the maximum, using a 3 min exposure time and 40× objective lens without binning. (1.31 MB TIF) [file pone.0010011.s007.tif]

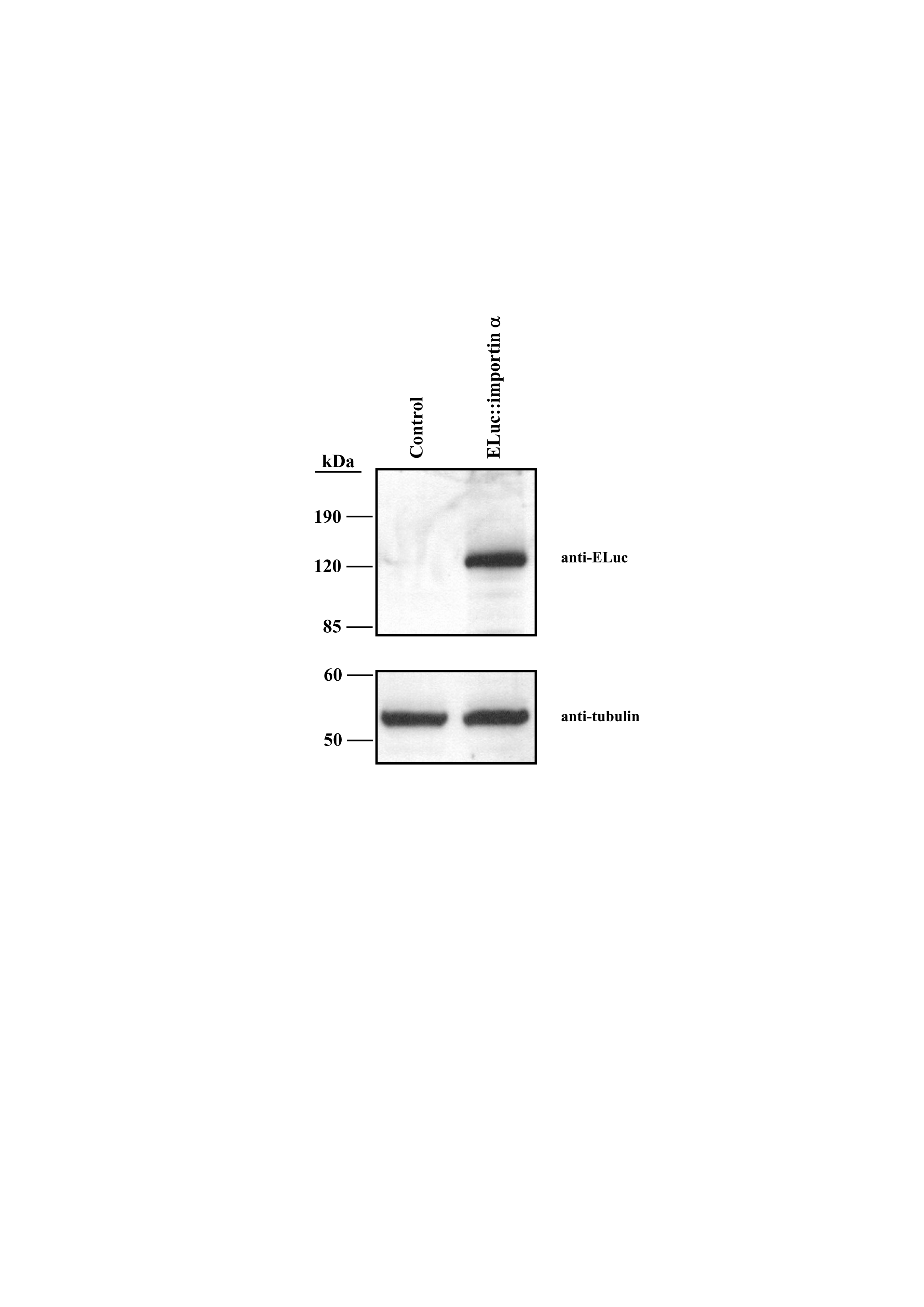

Supplement: Figure S8 — Western blot analysis of the ELuc::importinα fusion protein in NIH3T3 cells. NIH3T3 cells transfected with pCMV-ELuc::importin a were harvested and disrupted 48 h after transfection. The ELuc::importin Î± fusion protein was detected using an anti-ELuc antibody. Tubulin was used as an internal control. The positions of molecular weight markers are indicated on the left margin of each panel. (0.82 MB TIF) [file pone.0010011.s008.tif]
